# Design and synthesis of digitally encoded polymers that can be decoded and erased

**DOI:** 10.1038/ncomms8237

**Published:** 2015-05-26

**Authors:** Raj Kumar Roy, Anna Meszynska, Chloé Laure, Laurence Charles, Claire Verchin, Jean-François Lutz

**Affiliations:** 1 Precision Macromolecular Chemistry, Institut Charles Sadron, UPR22-CNRS, BP84047, 23 rue du Loess, 67034 Strasbourg Cedex 2, France; 2 Aix-Marseille Université – CNRS, UMR 7273, Institute of Radical Chemistry, 13397 Marseille Cedex 20, France

## Abstract

Biopolymers such as DNA store information in their chains using controlled sequences of monomers. Here we describe a non-natural information-containing macromolecule that can store and retrieve digital information. Monodisperse sequence-encoded poly(alkoxyamine amide)s were synthesized using an iterative strategy employing two chemoselective steps: the reaction of a primary amine with an acid anhydride and the radical coupling of a carbon-centred radical with a nitroxide. A binary code was implemented in the polymer chains using three monomers: one nitroxide spacer and two interchangeable anhydrides defined as 0-bit and 1-bit. This methodology allows encryption of any desired sequence in the chains. Moreover, the formed sequences are easy to decode using tandem mass spectrometry. Indeed, these polymers follow predictable fragmentation pathways that can be easily deciphered. Moreover, poly(alkoxyamine amide)s are thermolabile. Thus, the digital information encrypted in the chains can be erased by heating the polymers in the solid state or in solution.

## Abstract (web summary)

DNA and other biopolymers encode information based on a sequence of monomers. Here, the authors report a synthetic polymer capable of storing simple binary information which can subsequently be decoded by tandem mass spectroscopy, or permanently erased by heating.

## Graphical abstract

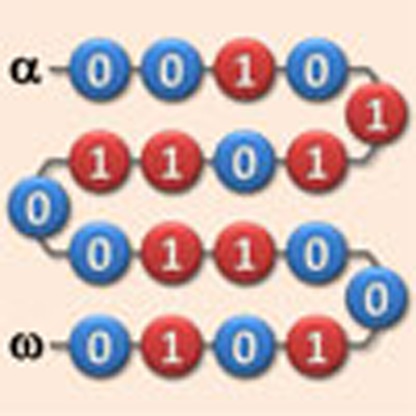

Synthetic polymers are today widely used in a large range of commodity and specialty products. Still, in most known applications, polymers are chosen for their unique physicochemical, structural and mechanical properties. However, in principle, synthetic polymers could exhibit more sophisticated molecular properties comparable to those of biopolymers. DNA, for example, stores information in its chains using a molecular code based on four different monomer units^1^. It was recently shown that artificial DNA oligomers can be used to store and retrieve a large amount of information^2^,^3^. However, DNA is not the only polymer allowing molecular encoding. In theory, any copolymer containing ordered co-monomer sequences could be used to store molecular information^4^,^5^,^6^,^7^. Indeed, a string of information can be implemented in a polymer using two co-monomers intentionally defined as 0-bit and 1-bit. However, reliable approaches for writing and reading molecular information in synthetic polymers are still missing^8^,^9^. Here we report a facile and rapid iterative method for preparing sequence-encoded polymers. This strategy relies on the use of two successive chemoselective coupling steps, that is, the reaction of a primary amine with a symmetric acid anhydride and the radical coupling of a carbon-centred radical with a nitroxide. This approach allows synthesis of monodisperse sequence-encoded poly(alkoxyamine amide)s. These polymers contain labile alkoxyamine linkages and are therefore easy to sequence using tandem mass spectrometry. A universal methodology for reading their encoded sequences is proposed in this work. Moreover, these polymers are thermolabile and therefore dynamic at temperatures above 60 °C. Thus, the molecular information encrypted in the chains can be erased by heating the polymers in the solid state or in solution.

## Results

### Chemoselective iterative synthesis of oligo(alkoxamine amide)s

**Figure 1:**
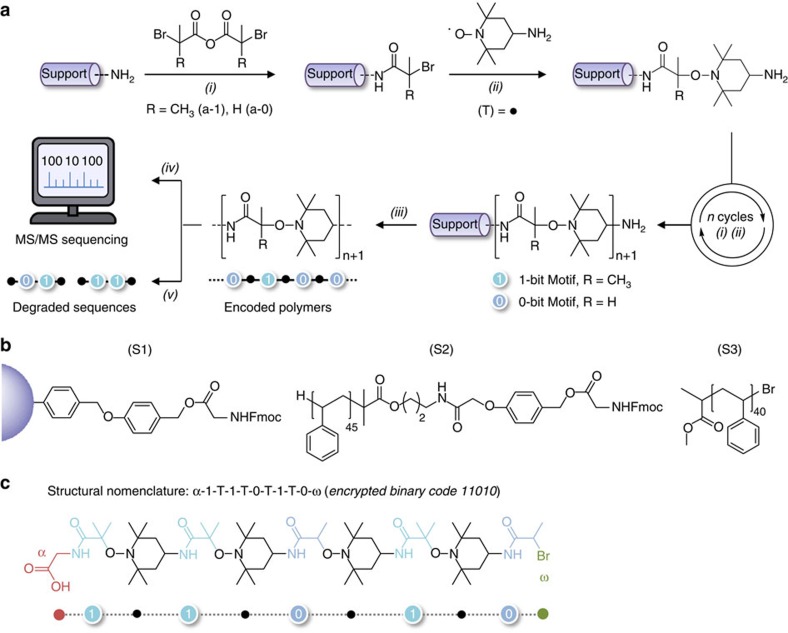
General concept for the synthesis of sequence-encodable polymers. (**a**) Strategy studied for the synthesis of oligo(alkoxyamine amide)s. This approach is based on two chemoselective reactions. The first *(i)* involves a primary amine and a symmetric bromo-functionalized anhydride, whereas the second *(ii)* is the radical coupling of a carbon-centred radical (obtained *in situ* by copper activation of an alkyl bromide) with an amino-functionalized nitroxide. Two symmetric anhydrides containing motifs defined as 0-bit and 1-bit can be used in an interchangeable manner in step *(i)* to create a binary code on the polymer chains. Experimental conditions: *(i)* THF, DIPEA or K_2_CO_3_, *(ii)* CuBr, Me_6_TREN, DMSO, *(iii)* TFA, CH_2_Cl_2_, *(iv)* ESI-MS/MS sequencing and *(v)* heating in the solid state or in solution. (**b**) Molecular structures of the solid-phase or soluble polymer supports used in this work. (**c**) Terminology and nomenclature used in the present work for the synthesis and sequencing of poly(alkoxyamine amide)s. The displayed example shows an oligomer containing the binary sequence 11010.

In order to store molecular information, synthetic polymers have to be monodisperse and exhibit controlled sequences of co-monomers^4^. Various strategies for preparing sequence-defined polymers have been reported^8^. For instance, monodisperse sequence-defined oligomers can be synthesized using template strategies^10^,^11^ or molecular machines^12^. However, the most-widespread approach relies on the stepwise coupling of monomer units on a solid support^13^. This approach is efficient but suffers from limitations: each coupling step should be nearly quantitative and defect-free; protecting groups are often required to control the reactivity of the monomers; and time-consuming cycles involving coupling, washing and deprotection steps are needed for each monomer attachment. While the former aspect is mandatory in iterative chemistry, the latter two can be avoided. Protecting groups are necessary for the synthesis of artificial biopolymers such as peptides and oligonucleotides but are not strictly required for the synthesis of non-natural oligomers, which can be achieved using chemoselective strategies^14^,^15^,^16^,^17^,^18^. Moreover, the duration of coupling cycles can be reduced by selecting fast reactions. For instance, the chemistry used in the present work is shown in Fig. 1a. It involves two different types of building blocks, each of them containing two kinds of reactive groups. The first building block comprises an acyclic symmetric acid anhydride and alkyl bromides, whereas the other contains a nitroxyl radical and a primary amine. Our concept is based on the fact that two chemoselective reactions can occur using these co-monomers. The first step is the reaction of a symmetric anhydride with a primary amine to form an *N*-substituted amide^19^. The second step is the coupling of a carbon-centred radical, obtained by copper activation of an alkyl bromide, with a nitroxide to afford an alkoxyamine. Such a radical–radical coupling reaction has been previously used for spin-trapping^20^ and alkoxyamine synthesis^21^. The alternating iterative use of the two coupling steps allows protecting-group-free synthesis of monodisperse oligomers on an amine- or bromine-functionalized solid support. An important advantage of this strategy is the fact that both coupling steps proceed in high yields within short time frames at room temperature. For instance, it is known that the radical–radical coupling of carbon-centred radicals with 2,2,6,6-tetramethylpiperidinyloxy (TEMPO) derivatives occur within a few minutes without noticeable by-product formation^22^. The reaction of an acid anhydride with a primary amine is also generally fast, but the obtained yields depend on experimental conditions. Supplementary Fig. 1 shows the characterization of model reactions, in which cyclohexylamine was reacted with the anhydride of either 2-bromo-isobutyric acid (**a-1**) or 2-bromopropionic acid (**a-0**). In the presence of K_2_CO_3_ and of an anhydride excess^23^, quantitative yields were obtained in a few minutes. Moreover, no imide by-products could be detected using mass spectrometry analysis.

**Figure 2:**
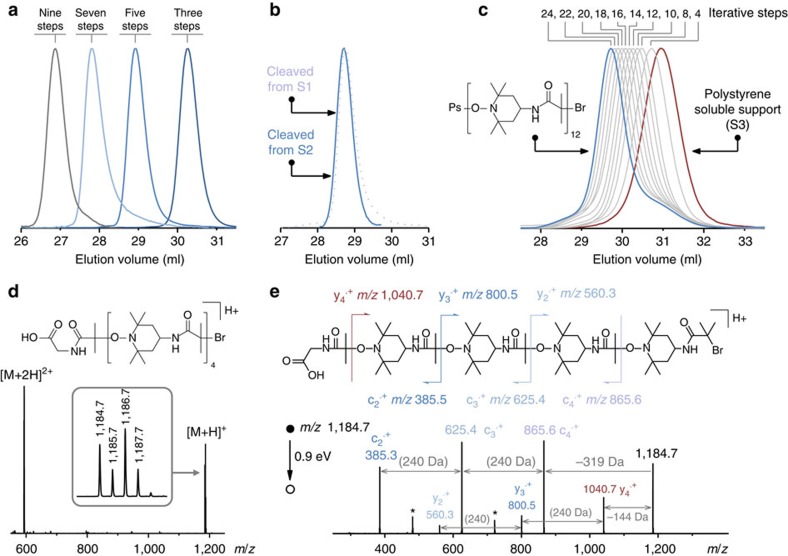
Characterization of oligo(alkoxyamine amide)s prepared using amino-TEMPO and 2-bromo-isobutyric anhydride. (**a**) SEC chromatograms recorded in THF for oligomers cleaved from support **S1** after three, five, seven and nine iterative steps. These chromatograms correspond to Entries, 2, 11, 13 and 16 in Supplementary Table 1. (**b**) SEC chromatograms recorded in THF for oligomers obtained after five iterative steps on the solid support **S1** (dotted purple line) or on the soluble support **S2** (full dark-blue line). These data correspond to Entries 2–3 in Supplementary Table 1. (**c**) SEC chromatograms recorded in THF for the iterative of oligo(alkoxyamine amide)s on the soluble support **S3**. The numbers listed above the chromatograms denote the number of iterative steps at which the analysis was performed. It should be noted that Fig. 2a,b was recorded on a SEC set-up for oligomer analysis, whereas Fig. 2c was recorded on a SEC set-up for high molecular weight analysis. Therefore, the displayed elution volumes are not comparable in all cases. (**d**) Positive mode ESI-MS spectrum recorded for an oligomer obtained after nine iterative steps on solid support **S1** (Supplementary Table 1, Entry 16). The inset shows the isotopic pattern of the [M+H]^+^ ion. (**e**) The ESI-MS/MS spectrum of an oligomer obtained after nine iterative steps on solid support **S1** (Supplementary Table 1, Entry 16). This MS/MS spectrum was obtained after collisional activation of the *m/z* 1,184.7 precursor ion containing the ^79^Br isotope. Stars indicate secondary fragments such as c_4_^+^−144 and c_3_^+^−144.

These preliminary considerations indicated that the two coupling steps shown in Fig. 1a are suitable for iterative solid-phase synthesis. In order to validate this experimentally, the alternating iterative oligomerization of amino-TEMPO (T) and **a-1** was first studied on a commercial glycine-loaded Wang resin (**S1**). Successive coupling steps were performed repeatedly on the resin. After reaching a given number of steps, the formed oligomers were cleaved from the solid support and analysed using mass spectrometry, size-exclusion chromatography (SEC) and NMR. Electrospray high-resolution mass spectrometry (ESI-HRMS) indicated that the formed oligomers are monodisperse (Supplementary Table 1 and Fig. 2d). This was also confirmed using the SEC analysis. Figure 2a shows the SEC chromatograms recorded for oligomers isolated after three, five, seven and nine iterative steps. In all cases, a sharp monomodal peak with a narrow molecular weight distribution (1.005<*M*_w_/*M*_n_<1.01) was observed. It should be noted that the apparent polydispersity index of monodisperse polymers is never equal to 1 in SEC because of axial dispersion and peak broadening^24^,^25^. Moreover, the molecular structure of the oligo(alkoxyamine amide)s was confirmed with NMR analysis (Supplementary Fig. 2 and Supplementary Fig. 3). It is interesting to note that, despite the fact that **1** generates a tertiary radical, the presence of unsaturated species due to H-transfer side reaction could not be detected in ^13^C NMR and was only marginally observed in ESI-HRMS^26^. These observations are in agreement with previous radical–radical coupling studies involving tertiary radicals, which indicate that this side reaction is prominent at elevated temperatures but disfavoured at milder temperatures^21^,^27^. On the whole, these results confirm that cycles involving successive amine–anhydride and radical–radical coupling steps are adequate for iterative synthesis. Still, it should be mentioned that, after performing a certain number of iterative steps (that is, from step 6 and beyond), incomplete yields (that is, ∼95%) were often observed for the radical coupling reaction when very short reaction times were used. However, this problem was easily solved by performing this reaction under microwave irradiation.

Besides conventional solid-phase chemistry, the oligo(alkoxyamine amide)s can also be easily prepared in other conditions. The iterative oligomerization of amino-TEMPO and **a-1** was also tested on polystyrene-based soluble supports. It is known that soluble polymer supports allow better reagent accessibility and sometimes higher yields than resins^28^. A glycine-loaded Wang soluble polystyrene support (**S2**) was tested for the synthesis of the oligo(alkoxyamine amide)s. It was found that this support allows a reliable synthesis of monodisperse alternating oligomers (Supplementary Table 1). As shown in Fig. 2b and Supplementary Fig. 4, oligomers of comparable quality could be obtained on **S1** and **S2**. The iterative chemistry was also investigated on a non-cleavable support (**S3**) prepared by atom transfer radical polymerization (ATRP)^29^. Owing to the mechanism of ATRP, **S3** is terminated by an alkyl bromide that can be used as initiator for the oligo(alkoxyamine amide) synthesis. Figure 2c shows a series of chromatograms obtained for a multistep synthesis on **S3**. It should be specified that these chromatograms were all recorded after performing the amine–anhydride step. Indeed, after the radical coupling step, the polymers possess a primary amine ω-chain end that interacts with SEC columns. A clean multistep growth can be observed in that figure. Each iteration resulted in an apparent molecular weight increase of ∼300–400 g mol^−1^ that roughly corresponds to the theoretical molecular weight that should be gained after performing one amine–anhydride step and one radical–radical coupling step (Supplementary Table 2). Comparable molecular weight increments were also observed on **S2** (Supplementary Fig. 4a). It is also important to note that the shape of the chromatograms does not change with the iterative growth (that is, the molecular weight distribution of the support-bound oligomer is similar to the one of the bare support). However, after 10 iteration steps on **S3**, a shoulder appears in the chromatograms at high elution volume. This peak is not due to the iterative process but corresponds most probably to the dead chains formed during the ATRP process^30^. Indeed, these chains do not contain any terminal halogen and therefore do no initiate multistep growth. Besides SEC, NMR monitoring confirmed the iterative attachment of T and **1** to **S3**. These results account for a smooth formation of oligo(alkoxyamine amide)s on the soluble support.

### Synthesis and MS/MS sequencing of binary-coded polymers

**Figure 3:**
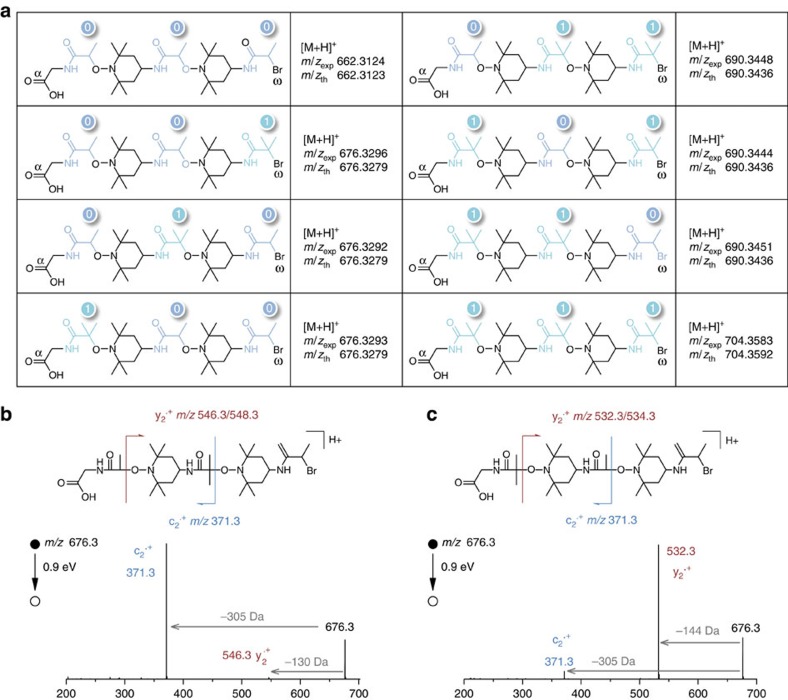
Synthesis and sequencing of sequence-encoded oligo(alkoxyamine amide)s. (**a**) ESI-HRMS characterization of sequence-encoded oligomers containing different binary triads synthesized on solid support **S1**. (**b**) ESI-MS/MS spectrum of a pentamer encoded with the binary triad 010 (Supplementary Table 1, Entry 6). (**c**) ESI-MS/MS spectrum of a pentamer encoded with the binary triad 100 (Supplementary Table 1, Entry 7). Spectra **b** and **c** were both derived from a precursor peak at *m/z* 676.3 corresponding to the ^79^Br isotope of the corresponding oligomer.

The chemistry shown in Fig. 1a was extended to the synthesis of sequence-encoded polymers. In order to encode a binary message in the polymer chains, three monomers were used: amino-TEMPO, **a-0** and **a-1**. The interchangeable use of **a-0** and **a-1** in the amine–anhydride step allows sequence-encoding. It should be noted that the nitroxide units could also be used to implement a code in the chains (that is, using interchangeable nitroxides in the radical coupling step). However, an anhydride-based language was chosen for the present work. At first, a series of eight model pentamers containing all possible binary triads was synthesized on **S1** (Fig. 3a). These oligomers were synthesized in five steps using three encoding building blocks and two nitroxide spacers. ESI-HRMS and SEC indicated the formation of monodisperse oligomers in all cases. It should be specified that, under similar experimental conditions, the moiety **0**-Br leads generally to lower yields than **1**-Br in the radical coupling step^21^. However, quantitative yields were obtained using an optimized amount of catalyst. Very interestingly, the sequence-encoded copolymers can be easily decoded. Various sequencing tools have been developed for proteomics and genomics during the past decades^9^. In particular, tremendous advances have been achieved in the field of biopolymers owing to tandem mass spectrometry (MS/MS) allowing their reliable, fast and automated sequencing based on universal dissociation rules^31^. Since MS/MS dissociation patterns strongly depend on the chemistry of the polymer backbone, no universal rules can however be envisaged for non-natural species. Instead, one has to refer to the specific fragmentation behaviour established for the targeted polymer family to decipher structural information from MS/MS spectra^32^. Using MS/MS, it was found here that oligo(alkoxyamine amide)s are remarkably easy to sequence. Owing to the lability of alkoxyamine linkages, there is no alternative dissociation reaction efficiently competing with the low-energy homolytic cleavage of those bonds between the TEMPO co-monomer and any **0** or **1** unit. Moreover, the nature of the coding units has no influence on this main fragmentation pathway. As a result, oligo(alkoxyamine amide)s dissociate into easy-to-read fragments (Supplementary Methods). In order to understand the basic principles of their sequencing, isomers containing different binary triads were first studied using MS/MS (Fig. 3b,c and Supplementary Figure 5). Depending on the analysed sequence, four fragments corresponding to the release of motifs α-**0** (−130 Da), α-**1** (−144 Da), ω-**0** (−305 Da) or ω-**1** (−319 Da) as radicals were easily identified in MS/MS spectra. For longer sequences, fragment series reveal the primary structure of the polymers (Fig. 2e and Supplementary Fig. 5f): intervals of 226 or 240 Da between α- or ω-containing fragments indicate the presence in the sequence of the motif T-**0** or T-**1**, respectively. The same fragmentation scenario was found in all analysed samples and can therefore be utilized for facile poly(alkoxyamine amide) sequencing. In order to illustrate the universality of this approach, a blind sequencing experiment was performed. A sequence-coded polymer (Supplementary Table 1, Entry 19) was synthesized and given for MS/MS analysis, but the sequence was not communicated to the MS experimenter. Nevertheless, the monomer-encoded ‘secret' message was easily deciphered.

### Thermal degradation of the coded polymers

**Figure 4:**
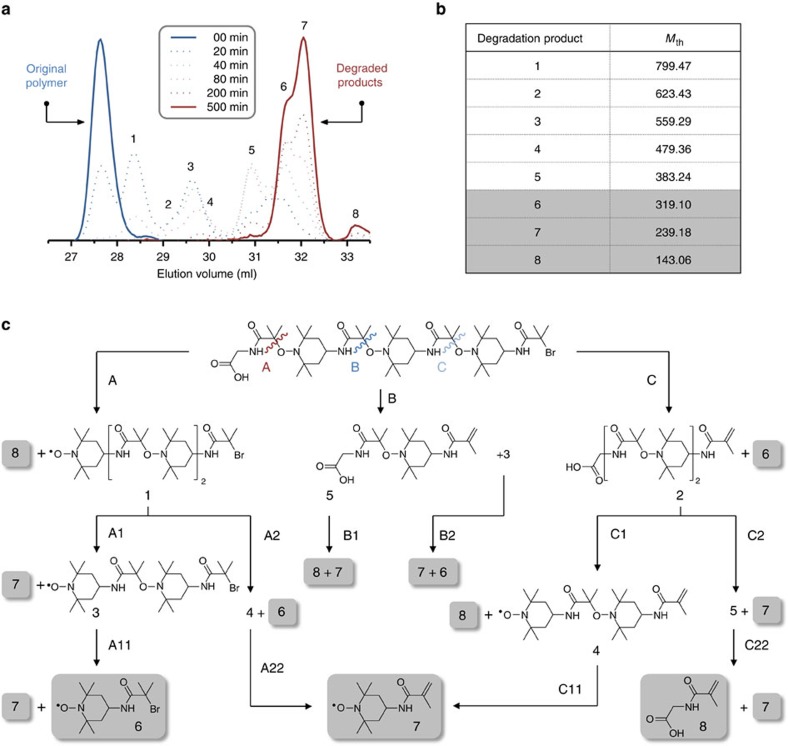
Controlled thermal degradation of a monodisperse heptamer performed in anisole solution at 120 °C and in the presence of a large excess of TEMPO. (**a**) Kinetic monitoring of the degradation process followed by SEC in THF. Dotted blue chromatograms show the early instant of the degradation process, whereas the dotted red chromatograms show later stages. (**b**) Theoretical molecular weight values (*M*_th_) of the degradation products that may be formed during the degradation process. The corresponding molecular formulae are shown in **c**. (**c**) Proposed degradation pathway. The structures highlighted in grey are final species. It should be noted that the hydroxylamines of 1, 3, 4, 6 and 7 may also be formed.

Furthermore, the formed polymers can be easily thermally modified. Indeed, polyalkoxyamines are thermolabile polymers that usually exhibit a dynamic behaviour when heated^33^,^34^. This is due to the homolysis of the C–ON bond that occurs at elevated temperatures^35^,^36^. For instance, C-TEMPO bonds dissociate, in general, above 60–70 °C (ref. 35). Interestingly, it was observed that sequence-encoded oligo(alkoxyamine amide) do reorganize in the solid state at 90 °C (Supplementary Fig. 6a). The thermal reorganization of these polymers was also studied in tetrahydrofuran (THF) solution at different temperatures. Above 75 °C, the polymers degrade into polydisperse samples. The speed of degradation can be modulated by temperature and proceed within a few hours above 120 °C (Supplementary Fig. 6b). SEC analysis indicated the formation of multimodal species that are formed by random carbon–carbon radical coupling and other side reactions. However, this degradation process can be more finely controlled. For instance, the probability of occurrence of carbon–carbon radical coupling can be reduced when a spin trap such as TEMPO is used in excess (Fig. 4). Under such conditions, a stepwise degradation process can be obtained (Fig. 4c). SEC analysis (Fig. 4a) indicated the formation of a controlled degradation cascade. Positive mode ESI-HRMS analysis of the final degradation products indicated the presence of the unsaturated degradation residues **6** and **7** and their corresponding hydroxylamines. Compound **8** was detected using negative mode ESI-HRMS. Overall, temperature can be used as a simple trigger to erase the sequence information stored in the polymers. It should be however noted that these materials are stable at room temperature. No sign of degradation was observed after several months of storage under standard laboratory conditions.

## Discussion

Binary-encoded poly(alkoxyamine amide)s have been identified as an interesting new class of synthetic polymers. These macromolecules permit to store a coded message that can be easily deciphered with MS/MS sequencing. In principle, a wide variety of other synthetic polymers could be used to store information^4^. However, sequence-coded poly(alkoxyamine amide)s exhibit important advantages that make them appealing for information-related applications. First of all, these polymers are easy to synthesize. As shown in the Results section, monodisperse sequence-defined poly(alkoxyamine amide)s can be prepared on different types of solid or soluble supports. Moreover, the protecting-group-free iterative approach introduced in this article is straightforward and permits to synthesize these polymers relatively rapidly. It should be also specified that the chain lengths reported here do not represent the upper limit of the approach. Longer polymers can be certainly prepared, in particular using automated robotic equipment. Still, it should be reminded that the efficiency of the coupling steps may decrease with increasing chain length and therefore that modified protocols (for example, using repeated steps or capping steps) might be needed to prepare longer polymers. Another important advantage of the methodology is the possibility to use different monomer codes. In this first proof-of-concept, a binary code based on anhydrides **a-0** and **a-1** was chosen. However, other monomer-based alphabets can be envisaged. For instance, chain encryption could be performed using other anhydrides or using a nitroxide-based code. The latter option is interesting since different nitroxides may lead to polymers containing C–ON bonds with different homolysis rates. Such a strategy would probably permit to tune the sequencing and erasing behaviour of the polymers. It should be also remarked that the concept is not restricted to binary codes, but could be extended to more complex monomer-based codes (for example, ternary or higher).

Sequence-encoded poly(alkoxyamine amide)s are also remarkably easy to sequence because of the presence of alkoxyamine ‘weak links' in their chains. These cleavable bonds enable an excellent ‘readability' in MS/MS sequencing. Our results show that synthetic polymers may be as easy—or even easier—to sequence than biopolymers. Indeed, the molecular structure of synthetic polymers can be optimized for a sequencing technology, whereas in biopolymer sequencing the read-out device has to be adapted to a molecular structure that is imposed by biology. In the present case, for example, the incorporation of ‘weak links' in the polymer simplify its MS/MS sequencing because no alternative dissociation reaction competes with the low-energy homolytic cleavage of the C–ON bond between a T-motif and any **0**/**1** motif. Moreover, the nature of the coding **0**/**1** units has no influence on the mechanism of this main fragmentation pathway. As a consequence, MS/MS data analysis is straightforward and can be performed in a few minutes. The analysis time can probably be further reduced with the help of a computer programme for analysing fragmentation data. Finally, it should be added that, while HRMS was used in this study for unambiguous assignment of product ions in order to establish their formation mechanism, the same sequencing results can be obtained using mass spectrometers operating with low-resolution mass analysers, such as triple quadrupole instruments that have become routine instruments in most laboratories.

The sequence-encoded polymers can also be thermally erased and degradation can be performed in the solid state or in solution. The proof-of-principle reported in this paper was provided by TEMPO-based polymers. However, as mentioned above, the thermal properties of poly(alkoxyamine amide)s (for example, storage half-life at room temperature and degradation rate at higher temperature) can be probably adjusted using other types of nitroxides. Moreover, it is imaginable to increase the storage half-life of these polymers by blending them in inorganic matrices^37^,^38^. These findings open up new application areas for synthetic polymers, for instance, in the fields of data storage, information processing and molecular identification. It is important to specify that these potential applications do not necessarily require very long polymer chains. For instance, a realistic, short-term, application for these coded polymers is the development of molecular barcodes for product identification. Binary-coded oligomers could be used for tagging high-value products in order to distinguish them from counterfeits. Altogether, the results shown in this article indicate that synthetic information-containing macromolecules may have a high relevance for manmade technologies.

## Methods

### Materials

4-Amino-2,2,6,6-tetramethylpiperidine-1-oxyl (4-amino-TEMPO, Tokyo Chemical Industry, 97%), 2,2,6,6-tetramethylpiperidine-1-oxyl (TEMPO, Alfa Aesar, 98%), 2-bromo-isobutyric acid (Alfa Aesar, 98%), 2-bromopropionic acid (Aldrich, 99%), tris(2-dimethylaminoethyl)amine (Alfa Aesar, >99%), methyl-2-bromopropionate (Aldrich, 98%), cyclohexamine (Aldrich, 99%), *N,N,N′,N′′,N′′*-pentamethyldiethylenetriamine (PMDETA, Aldrich, 99%), trifluoroacetic acid (Sigma-Aldrich, 99%), *N,N*-dicyclohexylcarbodiimide (Alfa Aesar, 99%), piperidine (Sigma-Aldrich, 99%), potassium carbonate (Prolabo, 99%), *N-*ethyldiisopropylamine (DIPEA, Alfa Aesar, 99%), dichloromethane (DCM, Carlo Erba, 99.9%), THF (Aldrich, 99%, stabilized with Butylated Hydroxytoluene), dimethyl sulfoxide (DMSO, Aldrich, >99.6%) were used as received. Copper-(I)-bromide (Sigma-Aldrich, 98%) was washed with glacial acetic acid in order to remove any soluble oxidized species, filtered, washed with ethanol and dried. 2-Bromoisobutyryl anhydride (**a-1**) was synthesized following a reported procedure^39^. The synthesis of 2-bromopropionic anhydride (**a**-**0**) is described in the Supplementary Methods. Fmoc-Gly-Wang resin (**S1**; 0.40–1.00 mmol g^−1^ loading) was purchased from Novabiochem/Merck. The cleavable Fmoc-Gly-Wang polystyrene-soluble support (**S2**) was prepared by ATRP and chain end modification as previously described (*M*_n_=5,700 g mol^−1^, *M*_w_/*M*_n_=1.10)^40^,^41^. The bromine-functional polystyrene-soluble support (**S3**) was prepared by ATRP in the presence of methyl-2-bromopropionate, copper-(I)-bromide and PMDETA (*M*_n_=4,000 g mol^−1^, *M*_w_/*M*_n_=1.11).

### Measurements and analysis

Two different SEC set-ups were used in this work. The first one was equipped with four PLGel Mixed C columns (5 μm, 30 cm, Ø=7.5 mm), a Wyatt Viscostar-II viscometer, a Wyatt TREOS light-scattering detector, a Shimadzu SPD-M20A diode array UV detector and a Wyatt Optilab T-rEX refractometer. This set-up was used for polymer characterization (1,000–3,000,000 g mol^−1^). The other set-up was equipped with a Shimadzu RiD-10 A refractometer, a Shimadzu SPD-10Avp UV detector and four monoporosity PLGel columns (5 μm, 30 cm, Ø=7.5 mm): 50, 100, 500 and 1,000 Å. This set-up was used for oligomer and short polymer characterization (100–20,000 g mol^−1^). In both set-ups, the mobile phase was THF with a flow rate of 1 ml min^−1^. Toluene was used as the internal reference. The calibration was based on linear PS standards from Polymer Laboratories. ^1^H NMR and ^13^C NMR spectra were recorded in CDCl_3_ on either a Bruker Avance 400 MHz or on a Bruker Avance 600 MHz spectrometers equipped with Ultrashield magnets. The 2D HSQC was realized using a Bruker 600 MHz spectrometer at 25 °C. The 2D HMBC was realized using a Bruker 400 MHz spectrometer at 25 °C. High-resolution ESI-HRMS and MS/MS experiments were performed using a QStar Elite mass spectrometer (Applied Biosystems SCIEX, Concord, ON, Canada) equipped with an ESI source operated in the positive mode. The capillary voltage was set at +5,500 V and the cone voltage at +75 V. In this hybrid instrument, ions were measured using an orthogonal acceleration time-of-flight mass analyser. In the MS mode, accurate mass measurements were performed using reference ions from a poly(propylene glycol) or a poly(ethylene glycol) internal standard. In the MS/MS mode, a quadrupole was used for selection of precursor ions to be further submitted to collision-induced dissociation in a collision cell. The precursor ion was used as the reference for accurate measurements of product ions in MS/MS spectra. In this instrument, air was used as the nebulizing gas (10 p.s.i.), while nitrogen was used as the curtain gas (20 p.s.i.) as well as collision gas. Instrument control, data acquisition and data processing were achieved using the Analyst software (QS 2.0) provided by Applied Biosystems. Oligomer solutions were prepared in MeOH supplemented with ammonium acetate (3 mM) and introduced in the ionization source with a syringe pump (flow rate: 5 μl min^−1^). Detailed information about the MS/MS sequencing methodology can be found in the Supplementary Methods.

### Synthesis of sequence-encoded oligo(alkoxyamine amide)s

The following examples describe the successive coupling of **1** (or **0**) and amino-TEMPO to the Fmoc-Gly-Wang resin **S1** and can be understood as a general procedure for the synthesis of oligo(alkoxyamine amide)s on a solid support. Steps B and C are interchangeable and should be alternated with step D. These steps can be repeated a certain number of times in order to reach a desired oligomer length. General procedures for synthesizing similar polymers on soluble supports **S2** and **S3** can be found in the Supplementary Methods. (A) Activation of **S1**: 0.1 g (0.079 mmol, 1 Eq.) of Fmoc-Gly-Wang resin **S1** (loading 0.79 mmol g^−1^) was used as a solid support and placed in a fritted plastic funnel. Before the iterative steps, the resin beads were swollen by gentle shaking in DCM for 0.5 h. Next, the Fmoc was removed by treatment with piperidine/DCM (2 ml/2 ml) for 10 min. The deprotection step was repeated a second time to ensure complete removal of the Fmoc-protecting groups from the resin beads. A Kaiser test made on few resin beads confirmed the presence of deprotected primary amine groups. (B) Attachment of the **1** motif to the resin: A mixture of **a-1** (0.1248, g, 5 Eq.) and K_2_CO_3_ (0.2 g, 18 Eq.) was added to a fritted funnel for solid-phase synthesis containing amino-functionalized resin beads. To the reaction mixture, 4 ml of THF was added and was shaken for 50 min on a mechanical shaker. After completion, the solution was drained out from the fritted funnel. The beads were washed with MeOH-H_2_O (1:1) to remove residual K_2_CO_3_ and afterwards with THF to remove the excess anhydride. (C) Attachment of the **0**-motif to the resin: A solution of **a-0** (0.1248, g, 5.3 Eq.) in THF (4 ml) was added along with DIPEA (0.25 ml, 18 Eq.) to a fritted funnel for solid-phase synthesis containing amino-functionalized resin beads. The mixture was shaken for 50 min on a mechanical shaker. After reaction, the solution was drained out from the fritted funnel. The beads were washed several times with THF. The use of DIPEA as a base is recommended for this step because it was observed that K_2_CO_3_ forms an inhomogeneous gel with **a-0** in THF. (D) Attachment of amino-TEMPO to the resin: Amino-TEMPO (0.08 g, 6 Eq.) and Me_6_TREN (0.078 ml, 3.3 Eq.) were dissolved in 4 ml of anhydrous DMSO and were placed into a fritted funnel containing bromide-functionalized resin beads. The funnel was sealed with a rubber septum and the reaction mixture was purged with argon for ∼10 min. Then, CuBr (0.034 g, 3 Eq.) was rapidly added. The mixture was shaken for 25 min under inert atmosphere. After reaction, the solution was drained out from the fritted funnel. The beads were washed several times with THF. It is important to mention that the efficiency of this reaction is somehow reduced after performing several iterations on the resin. Thus, from the sixth step and beyond, a microwave synthesizer (CEM Liberty 1TM, Saclay, France) was used for increasing the yields of radical coupling. Typically, the reactions were irradiated at 150 W of microwave power at 40 °C for ∼90 min. (E) Cleavage of the oligomers from the resin: Cleavage of the poly(alkoxyamine amide)s from the resin was performed in TFA/DCM solution (1/1) for 2 h. After reaction, the solution was filtered, concentrated and precipitated in cold diethylether. The precipitate was filtered and redissolved in THF, and small amounts of insoluble resin fragments were separated by filtration. The filtrate was concentrated and the oligomers were isolated from cold diethylether precipitation. Trimers cannot be precipitated and were isolated by removing solvent and TFA. Example of final yield is (heptamer) 39 mg from 100 mg of **S1**; yield=52%.

### Thermal degradation of the oligomers

For solid state degradation tests, the oligo(alkoxyamine amide) was dissolved in a small amount of THF. The solution was poured on a glass slide and the THF was evaporated. The formed thin film was placed in a heating oven at 90 °C for several hours. For degradation tests in solution, the oligo(alkoxyamine amide) was dissolved in anisole and heated for 24 h under inert atmosphere at different temperatures, that is 50, 75, 100, 120 and 125 °C. For solution degradation tests in the presence of TEMPO, a poly(alkoxyamine amide) (Entry 14 in Supplementary Table 1, 20 mg, 1 Eq.) and TEMPO (39.6 mg, 12 Eq.) were dissolved in anisole and heated at 120 °C for several hours under argon atmosphere. In all experiments, the polymer degradation was monitored with SEC.

## Additional information

**How to cite this article**: Roy, R. K. *et al.* Design and synthesis of digitally encoded polymers that can be decoded and erased. *Nat. Commun.* 6:7237 doi: 10.1038/ncomms8237 (2015).

## Supplementary Material

Supplementary Information Supplementary Figures 1-6, Supplementary Tables 1-2, Supplementary Methods and Supplementary References

## References

[b1] Ecker J. R. *et al.* Genomics: ENCODE explained. Nature 489, 52–55 (2012). 22955614 10.1038/489052a

[b2] Church G. M., Gao Y. & Kosuri S. Next-generation digital information storage in DNA. Science 337, 1628–1628 (2012). 22903519 10.1126/science.1226355

[b3] Goldman N. *et al.* Towards practical, high-capacity, low-maintenance information storage in synthesized DNA. Nature 494, 77–80 (2013). 23354052 10.1038/nature11875 PMC3672958

[b4] Colquhoun H. & Lutz J.-F. Information-containing macromolecules. Nat. Chem. 6, 455–456 (2014). 24848219 10.1038/nchem.1958

[b5] Andrieux D. & Gaspard P. Nonequilibrium generation of information in copolymerization processes. Proc. Natl Acad. Sci. USA 105, 9516–9521 (2008). 18606997 10.1073/pnas.0802049105 PMC2474480

[b6] Zhu Z., Cardin C. J., Gan Y. & Colquhoun H. M. Sequence-selective assembly of tweezer molecules on linear templates enables frameshift-reading of sequence information. Nat. Chem. 2, 653–660 (2010). 20651728 10.1038/nchem.699

[b7] Trinh T. T., Oswald L., Chan-Seng D. & Lutz J.-F. Synthesis of molecularly encoded oligomers using a chemoselective ‘AB+CD' iterative approach. Macromol. Rapid Commun. 35, 141–145 (2014). 24338828 10.1002/marc.201300774

[b8] Lutz J.-F., Ouchi M., Liu D. R. & Sawamoto M. Sequence-controlled polymers. Science 341, 1238149 (2013). 23929982 10.1126/science.1238149

[b9] Mutlu H. & Lutz J.-F. Reading polymers: sequencing of natural and synthetic macromolecules. Angew. Chem. Int. Ed. 53, 13010–13019 (2014). 10.1002/anie.201406766 25283068

[b10] McKee M. L. *et al.* Multistep DNA-templated reactions for the synthesis of functional sequence controlled oligomers. Angew. Chem. Int. Ed. 49, 7948–7951 (2010). 10.1002/anie.201002721 20836102

[b11] Niu J., Hili R. & Liu D. R. Enzyme-free translation of DNA into sequence-defined synthetic polymers structurally unrelated to nucleic acids. Nat. Chem. 5, 282–292 (2013). 23511416 10.1038/nchem.1577 PMC4277153

[b12] Lewandowski B. *et al.* Sequence-specific peptide synthesis by an artificial small-molecule machine. Science 339, 189–193 (2013). 23307739 10.1126/science.1229753

[b13] Merrifield R. B. Solid Phase Synthesis (Nobel Lecture). Angew. Chem. Int. Ed. 24, 799–810 (1985).

[b14] Zuckermann R. N., Kerr J. M., Kent S. B. H. & Moos W. H. Efficient method for the preparation of peptoids [oligo(N-substituted glycines)] by submonomer solid-phase synthesis. J. Am. Chem. Soc. 114, 10646–10647 (1992).

[b15] Pfeifer S., Zarafshani Z., Badi N. & Lutz J.-F. Liquid-phase synthesis of block copolymers containing sequence-ordered segments. J. Am. Chem. Soc. 131, 9195–9197 (2009). 19522508 10.1021/ja903635y

[b16] Espeel P. *et al.* Multifunctionalized sequence-defined oligomers from a single building block. Angew. Chem. Int. Ed. 52, 13261–13264 (2013). 10.1002/anie.201307439 24174322

[b17] Solleder S. C. & Meier M. A. R. Sequence control in polymer chemistry through the passerini three-component reaction. Angew. Chem. Int. Ed. 53, 711–714 (2014). 10.1002/anie.201308960 24307280

[b18] Porel M. & Alabi C. A. Sequence-defined polymers via orthogonal allyl acrylamide building blocks. J. Am. Chem. Soc. 136, 13162–13165 (2014). 25204618 10.1021/ja507262t

[b19] Naik S., Bhattacharjya G., Talukdar B. & Patel B. K. Chemoselective acylation of amines in aqueous media. Eur. J. Org. Chem. 2004, 1254–1260 (2004).

[b20] Rizzardo E. & Solomon D. A new method for investigating the mechanism of initiation of radical polymerization. Polym. Bull. 1, 529–534 (1979).

[b21] Matyjaszewski K., Woodworth B. E., Zhang X., Gaynor S. G. & Metzner Z. Simple and Efficient synthesis of various alkoxyamines for stable free radical polymerization. Macromolecules 31, 5955–5957 (1998).

[b22] Kulis J., Bell C. A., Micallef A. S., Jia Z. & Monteiro M. J. Rapid, selective, and reversible nitroxide radical coupling (nrc) reactions at ambient temperature. Macromolecules 42, 8218–8227 (2009).

[b23] Yang H., Goyal N., Ella-Menye J. R., Williams K. & Wang G. Synthesis of chiral five-, six-, and seven-membered heterocycles from (S)-3-Hydroxy-γ-butyrolactone. Synthesis (Mass) 2012, 561–568 (2012).

[b24] Marais L., Gallot Z. & Benoît H. A new method for correcting axial dispersion in GPC. J. Appl. Polym. Sci. 21, 1955–1964 (1977).

[b25] Striegel A. M., Yau W. W., Kirkland J. J. & Bly D. B. in Modern Size-Exclusion Liquid Chromatography Ch. **3** 49–91John Wiley & Sons (2009).

[b26] Gryn'ova G., Lin C. Y. & Coote M. L. Which side-reactions compromise nitroxide mediated polymerization? Polym. Chem. 4, 3744–3754 (2013).

[b27] Lin W., Huang B., Fu Q., Wang G. & Huang J. Investigation of nitroxide radical coupling reaction in wide temperature range and different catalyst system. J. Polym. Sci. 48, 2991–2999 (2010).

[b28] Bayer E. & Mutter M. Liquid phase synthesis of peptides. Nature 237, 512–513 (1972). 12635201 10.1038/237512a0

[b29] Patten T. E., Xia J., Abernathy T. & Matyjaszewski K. Polymers with very low polydispersities from atom transfer radical polymerization. Science 272, 866–868 (1996). 8662578 10.1126/science.272.5263.866

[b30] Lutz J.-F. & Matyjaszewski K. Nuclear magnetic resonance monitoring of chain-end functionality in the atom transfer radical polymerization of styrene. J. Polym. Sci. 43, 897–910 (2005).

[b31] Biemann K. Laying the groundwork for proteomics: Mass spectrometry from 1958 to 1988. Int. J. Mass Spectrom. 259, 1–7 (2007). 10.1016/j.jprot.2014.01.008 24448399

[b32] Wesdemiotis C. *et al.* Fragmentation pathways of polymer ions. Mass Spectrom. Rev. 30, 523–559 (2011). 20623599 10.1002/mas.20282

[b33] Maeda T., Otsuka H. & Takahara A. Dynamic covalent polymers: Reorganizable polymers with dynamic covalent bonds. Prog. Polym. Sci. 34, 581–604 (2009).

[b34] Lehn J.-M. Dynamers: dynamic molecular and supramolecular polymers. Prog. Polym. Sci. 30, 814–831 (2005).

[b35] Marque S., Le Mercier C., Tordo P. & Fischer H. Factors influencing the C−O−bond homolysis of trialkylhydroxylamines. Macromolecules 33, 4403–4410 (2000).

[b36] Bertin D., Gigmes D., Marque S. R. A. & Tordo P. Polar, steric, and stabilization effects in alkoxyamines C−ON bond homolysis: a multiparameter analysis. Macromolecules 38, 2638–2650 (2005).

[b37] Kaplan M. DNA has a 521-year half-life. Nat. News doi:10.1038/nature.2012.11555 (2012).

[b38] Grass R. N., Heckel R., Puddu M., Paunescu D. & Stark W. J. Robust chemical preservation of digital information on DNA in silica with error-correcting codes. Angew. Chem. Int. Ed. 54, 2552–2555 (2015). 10.1002/anie.201411378 25650567

[b39] Östmark E., Harrisson S., Wooley K. L. & Malmström E. E. Comb polymers prepared by ATRP from hydroxypropyl cellulose. Biomacromolecules 8, 1138–1148 (2007). 17367185 10.1021/bm061043w

[b40] Meszynska A., Badi N., Borner H. G. & Lutz J.-F. ‘Inverse' synthesis of polymer bioconjugates using soluble supports. Chem. Commun. 48, 3887–3889 (2012). 10.1039/c2cc30233k 22410574

[b41] Oswald L., Trinh T. T., Chan-Seng D. & Lutz J.-F. Debromination of ATRP-made Wang soluble polymer supports. Polymer. (Guildf). doi:10.1016/j.polymer.2015.1002.1057.

