# Supplementary material for: Design and synthesis of digitally encoded polymers that can be decoded and erased
Source: Nat Commun. 2015 May 26;6:7237. doi: 10.1038/ncomms8237 (PMC4455100; doi:10.1038/ncomms8237)
Supplement: Supplementary Information — Supplementary Figures 1-6, Supplementary Tables 1-2, Supplementary Methods and Supplementary References [file ncomms8237-s1.pdf]

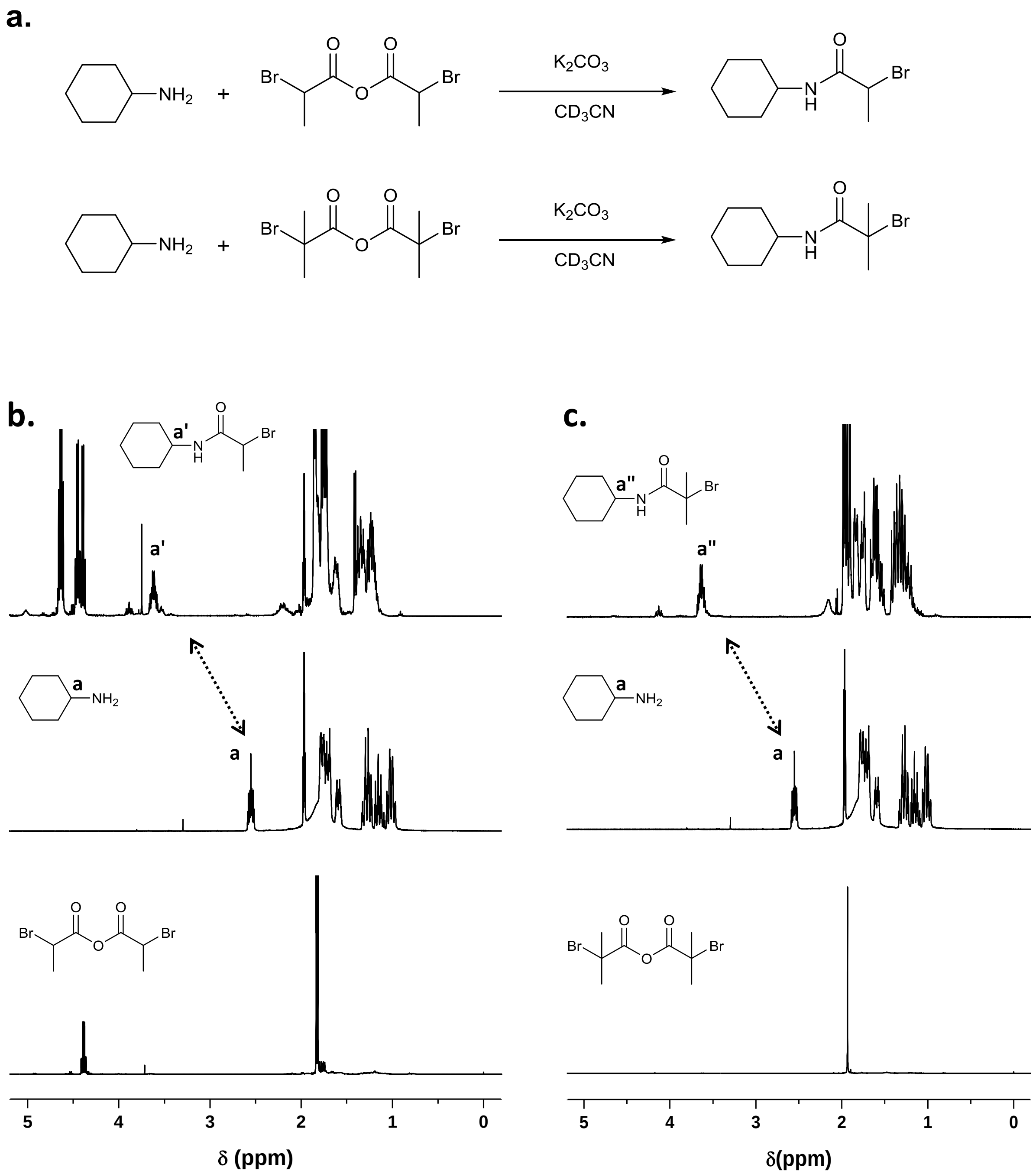

Supplementary Figure 1. Model reactions. (**a**) Model primary amine-anhydride coupling reactions performed in an NMR tube at room temperature (see supplementary methods). (**b**) ^{1}H NMR characterization of the model reaction involving cyclohexylamine and 2-bromopropionyl anhydride. (**c**) ^{1}H NMR characterization of the model reaction involving cyclohexylamine and 2-bromoisobutyryl anhydride.

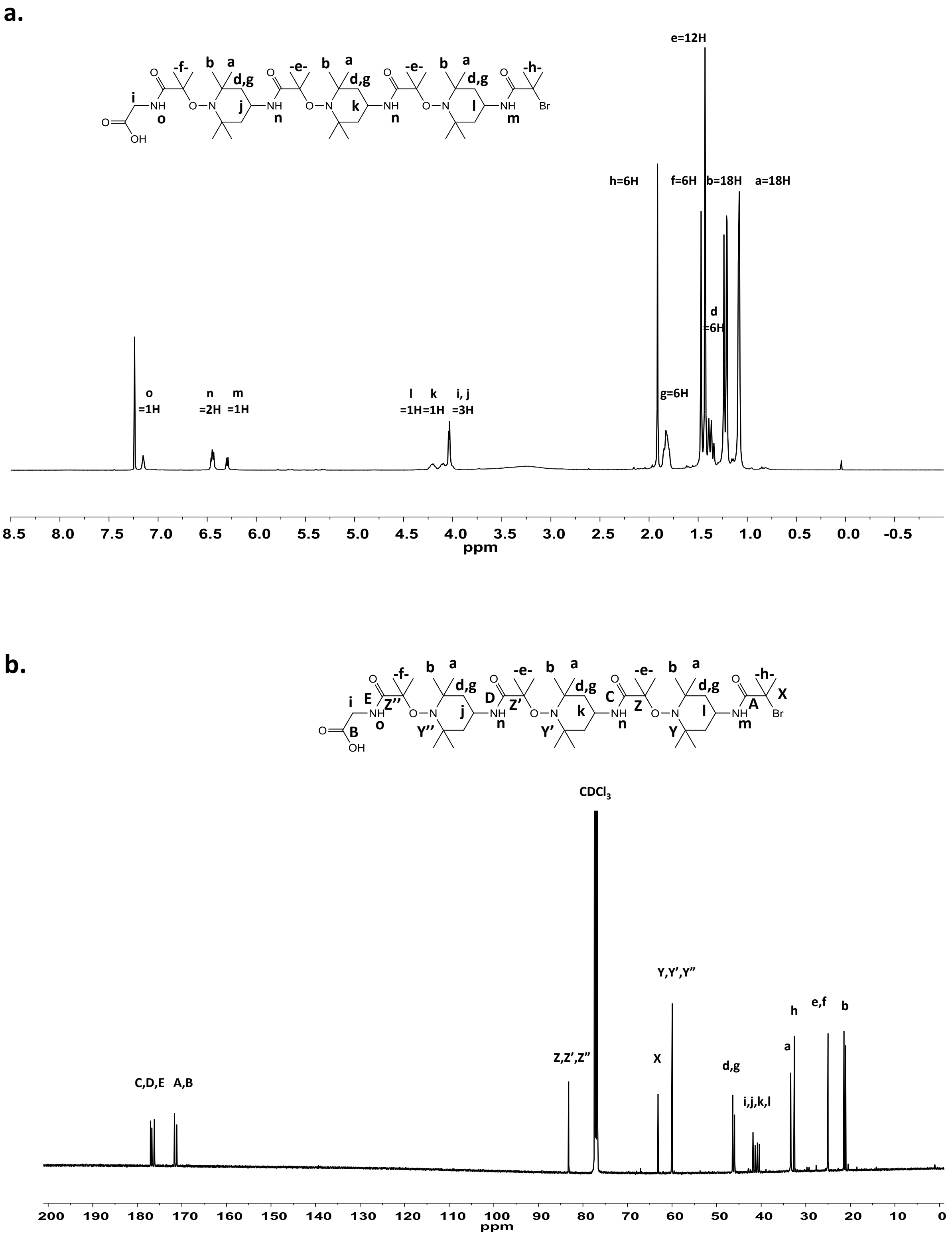

Supplementary Figure 2. NMR characterization of an oligo(alkoxyamine amide) prepared on S1. The displayed spectra correspond to Entry 13 in Supplementary Table 1. (**a**) ^{1}H NMR recorded in $CDCl_3$. (**b**) ^{13}C NMR recorded in $CDCl_3$.

a.

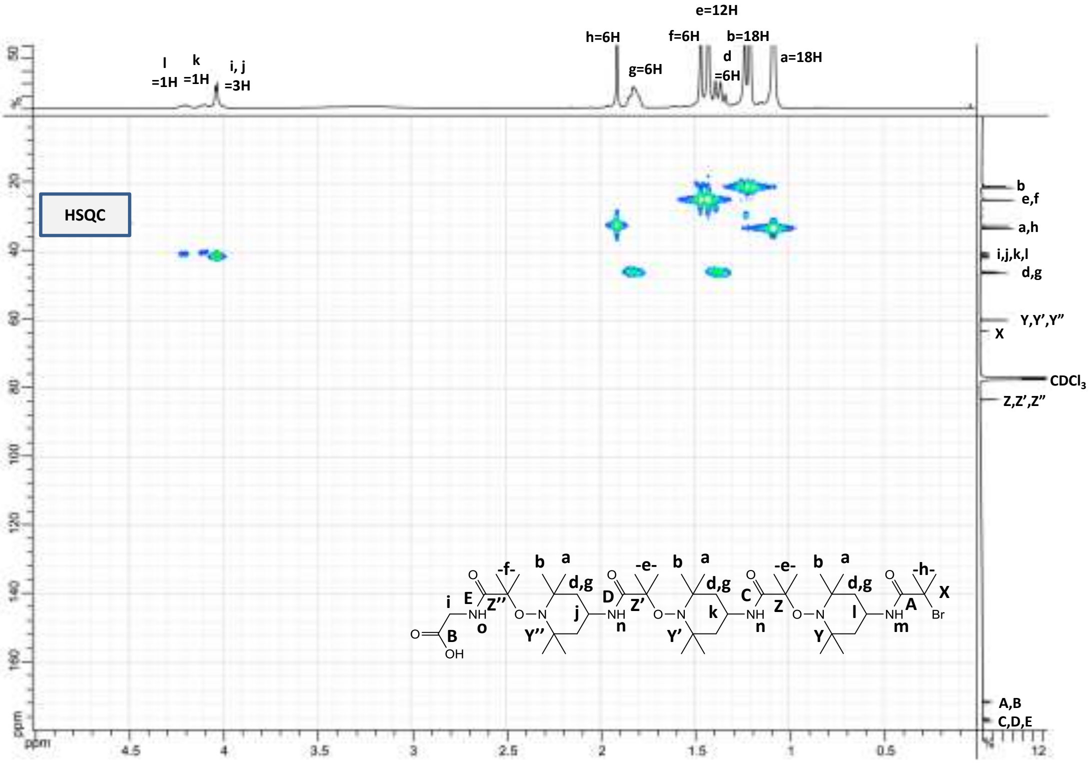

b.

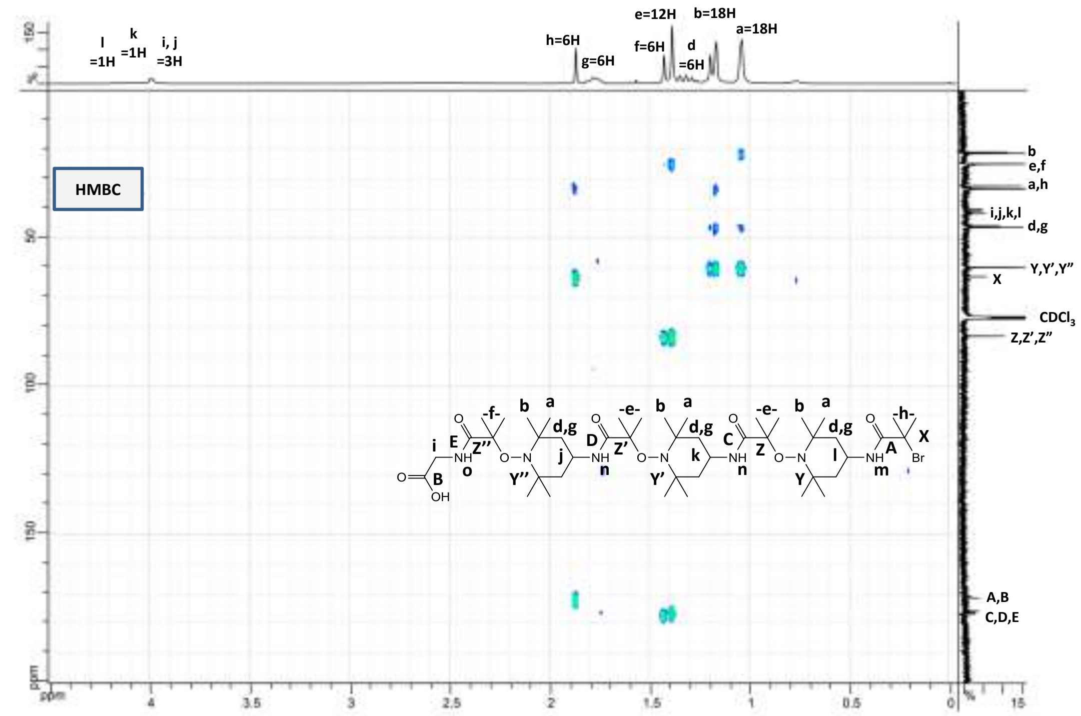

Supplementary Figure 3. 2D NMR characterization of an oligo(alkoxyamine amide) prepared on S1. The displayed spectra correspond to Entry 13 in Supplementary Table 1. (**a**) 2D (^{1}H, ^{13}C) HSQC recorded in $CDCl_3$. (**b**) 2D (^{1}H, ^{13}C) HMBC recorded in $CDCl_3$.

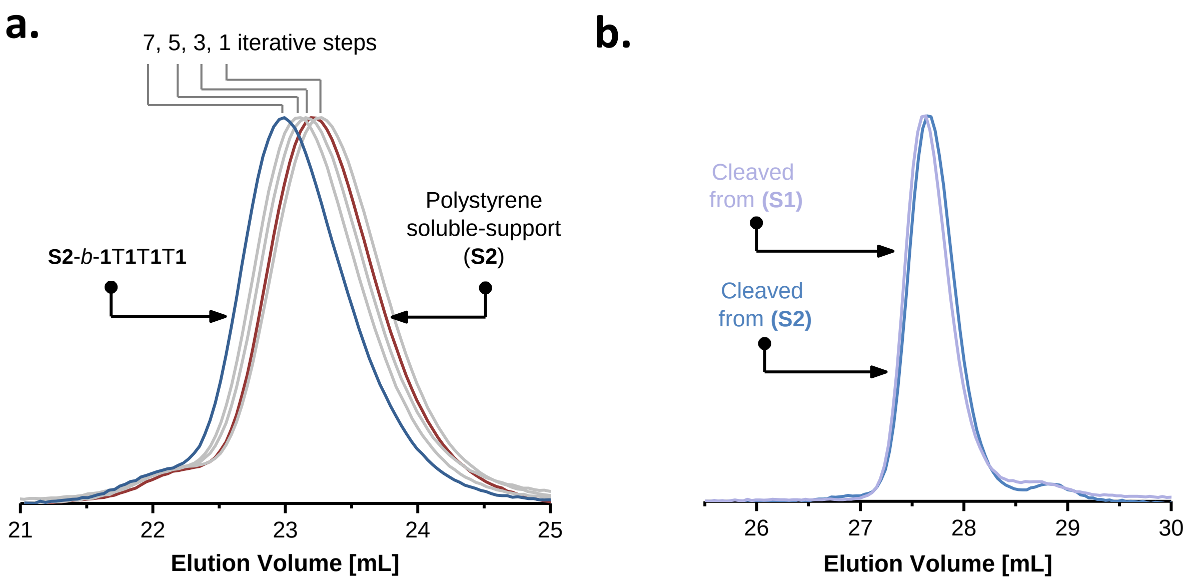

Supplementary Figure 4. Oligomer synthesis on S2. (**a**) SEC chromatograms recorded in THF for the multistep growth of a poly(alkoxyamine amide) on soluble support **S2**. The numbers listed above the chromatograms denote the number of iterative steps at which the analysis was done. (**b**) SEC chromatograms recorded in THF for oligomers obtained after 7 iterative steps on the solid support **S1** (purple line) or on the soluble support **S2** (dark-blue line). These data correspond to Entries 13-14 in Supplementary Table 1.

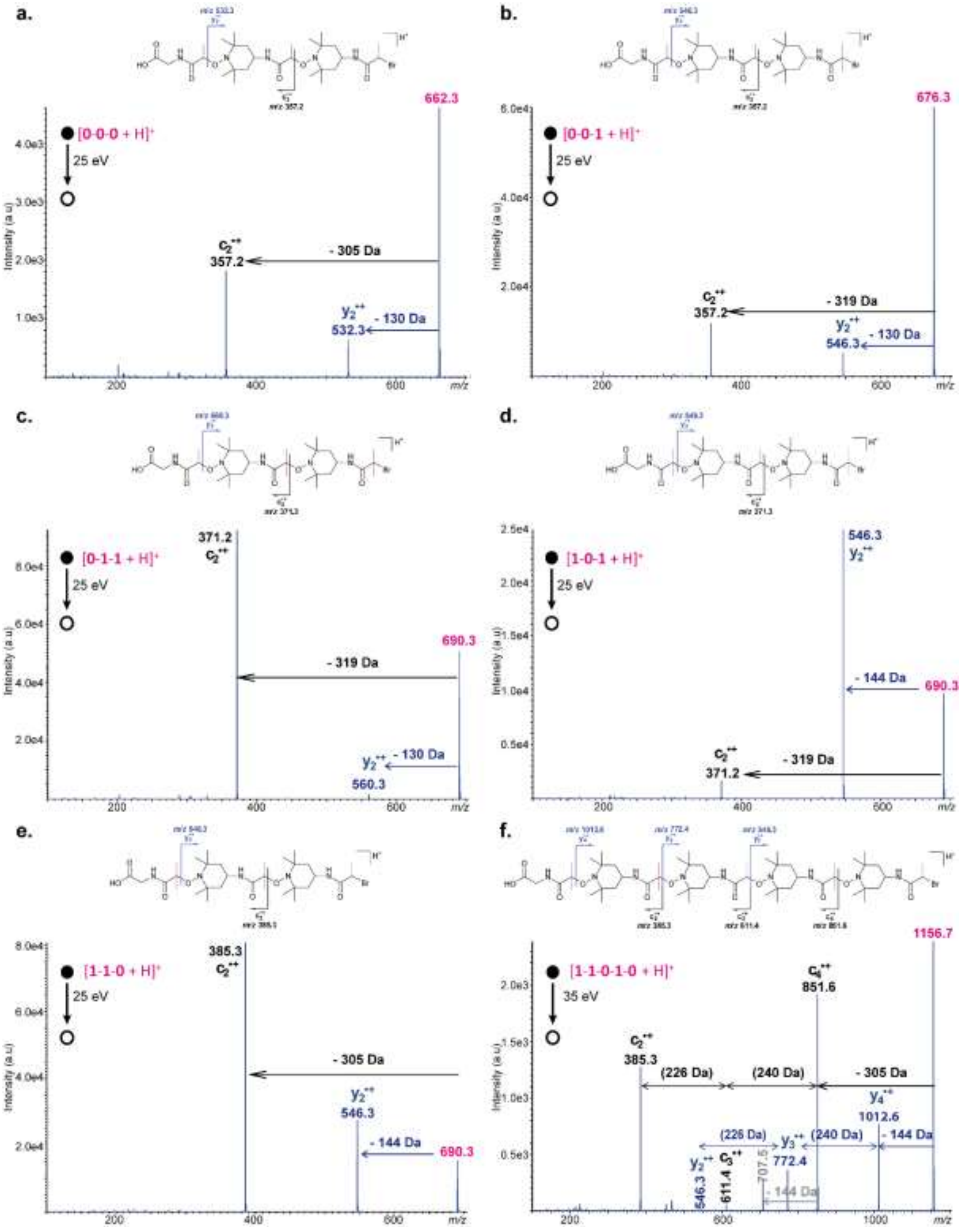

Supplementary Figure 5. Oligo(alkoxyamine amide)s sequencing. ESI-MS/MS spectra recorded for (**a**) Oligomer 000 (Supplementary Table 1, Entry 4), (**b**) Oligomer 001 (Supplementary Table 1, Entry 5), (**c**) Oligomer 011 (Supplementary Table 1, Entry 8), (**d**) Oligomer 101 (Supplementary Table 1, Entry 9), (**e**) Oligomer 110 (Supplementary Table 1, Entry 10), (**f**) Oligomer 11010 (Supplementary Table 1, Entry 18).

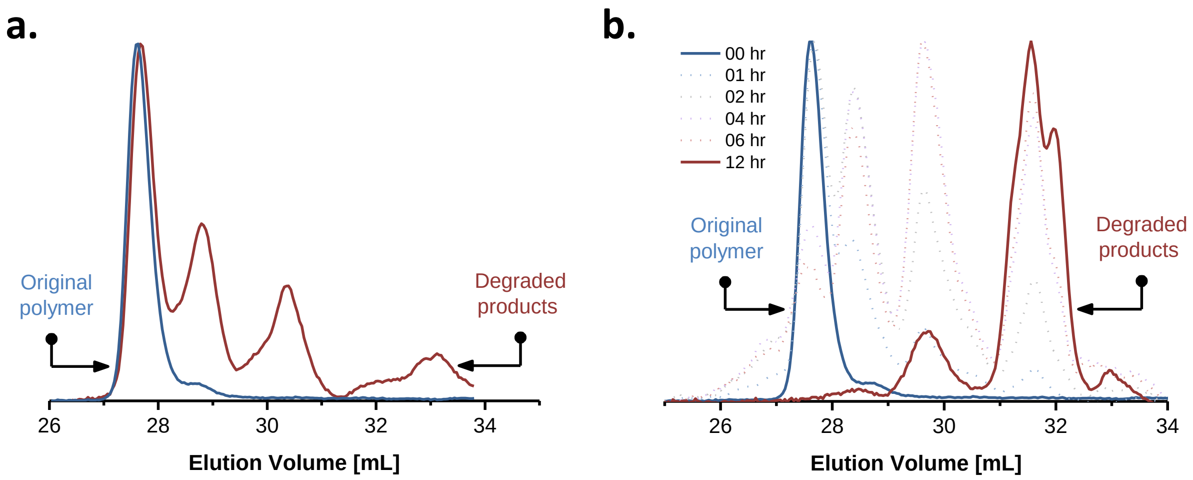

Supplementary Figure 6. Thermal degradation of oligo(alkoxyamine amide)s. (**a**) SEC chromatograms recorded before (blue) and after 12 hours of thermal treatment at 90°C in the solid-state. (**a**) SEC kinetic monitoring of the thermal degradation of a monodisperse heptamer performed in anisole solution at 120°C. Dotted blue chromatograms show the early instant of the degradation process, whereas the dotted red chromatograms show later stages.

Supplementary Table 1. Characterization of sequence-encoded poly(alkoxyamine amide)s synthesized on supports **S1** and **S2**.

	Sequence[a]	Code	S	m/z_{th}[b]	m/z_{exp}[b]	M_p[c]	M_n[c]	M_w/M_n[c]
**1**	α-**0**-T-**0**-ω	00	S1	436.1442	-	534	528	1.003
**2**	α-**1**-T-**1**-ω	11	S1	464.1755	464.1756	510	465	1.005
**3**	α-**1**-T-**1**-ω	11	S2	464.1755	464.1752	501	483	1.006
**4**	α-**0**-T-**0**-T-**0**-ω	000	S1	662.3123	662.3124	681	660	1.002
**5**	α-**0**-T-**0**-T-**1**-ω	001	S1	676.3279	676.3296	699	681	1.007
**6**	α-**0**-T-**1**-T-**0**-ω	010	S1	676.3279	676.3292	691	656	1.007
**7**	α-**1**-T-**0**-T-**0**-ω	100	S1	676.3279	676.3293	692	634	1.010
**8**	α-**0**-T-**1**-T-**1**-ω	011	S1	690.3436	690.3448	683	639	1.009
**9**	α-**1**-T-**0**-T-**1**-ω	101	S1	690.3436	690.3444	709	674	1.007
**10**	α-**1**-T-**1**-T-**0**-ω	110	S1	690.3436	690.3451	685	639	1.012
**11**	α-**1**-T-**1**-T-**1**-ω	111	S1	704.3592	704.3583	686	683	1.006
**12**	α-**1**-T-**1**-T-**1**-ω	111	S2	704.3592	-	783	771	1.002
**13**	α-**1**-T-**1**-T-**1**-T-**1**-ω	1111	S1	944.5430	944.5417	956	930	1.010
**14**	α-**1**-T-**1**-T-**1**-T-**1**-ω	1111	S2	944.5430	944.5427	950	933	1.012
**15**	α-**0**-T-**0**-T-**0**-T-**0**-ω	0000	S1	888.4804	-	1020	957	1.019
**16**	α-**1**-T-**1**-T-**1**-T-**1**-T-**1**-ω	11111	S1	1184.7268	1184.7264	1191	1146	1.010
**17**	α-**0**-T-**0**-T-**0**-T-**1**-T-**1**-ω	00011	S1	1141.6798	-	1270	1202	1.040
**18**	α-**1**-T-**1**-T-**0**-T-**1**-T-**0**-ω	11010	S1	1156.6955	1156.6958	1199	1001	1.060
**19**	α-**1**-T-**0**-T-**1**-T-**1**-T-**0**-ω	10110	S1	1156.6955	1156.6956	-	-	-
**20**	α-**1**-(T-**1**)$_6$-ω	1111111	S1	834.0508	834.0515	1977	1740	1.030

[a] The Greek letters α and ω denote the chain-ends of the polymer. The letter T denotes the TEMPO spacer. The numbers **0** and **1** correspond to the building-blocks defined as 0-bit or 1-bit. [b] Theoretical and experimental *m/z* values found by ESI-HRMS for the monoisotopic peak of the protonated molecules $[M+H]^+$. For Entry 20, values corresponding to the doubly protonated molecule $[M+2H]^{2+}$ are presented. [c] Values measured by size exclusion chromatography. M_n is the number average molecular weight. M_p is the peak molecular weight. M_w/M_n is the molecular weight distribution.

Supplementary Table 2. Characterization of sequence-encoded poly(alkoxyamine amide)s synthesized on support **S3**.

	**Sequence**[a]	M_p[b]	ΔM_p[c]
**1**	S3	4000	-
**2**	S3-*b*-T**1**T**1**	4856	856
**3**	S3-*b*-T**1**T**1**T**1**T**1**	5519	663
**4**	S3-*b*-T**1**T**1**T**1**T**1**T**1**	6017	498
**5**	S3-*b*-T**1**T**1**T**1**T**1**T**1**T**1**	6341	324
**6**	S3-*b*-T**1**T**1**T**1**T**1**T**1**T**1**T**1**	6673	332
**7**	S3-*b*-T**1**T**1**T**1**T**1**T**1**T**1**T**1**T**1**	7307	634
**8**	S3-*b*-T**1**T**1**T**1**T**1**T**1**T**1**T**1**T**1**T**1**	7674	367
**9**	S3-*b*-T**1**T**1**T**1**T**1**T**1**T**1**T**1**T**1**T**1**T**1**	8067	393
**10**	S3-*b*-T**1**T**1**T**1**T**1**T**1**T**1**T**1**T**1**T**1**T**1**T**1**	8477	410
**11**	S3-*b*-T**1**T**1**T**1**T**1**T**1**T**1**T**1**T**1**T**1**T**1**T**1**T**1**	9009	532

[a] The letter T denotes the TEMPO spacer. The number **1** corresponds to the building-blocks defined as 1-bit. [b] Values measured by size exclusion chromatography. M_p is the peak molecular weight. [c] $\Delta M_p = M_{p,\ Entry\ n} - M_{p,\ Entry\ n-1}$.

Supplementary methods.

Synthesis of 2-bromopropionic anhydride (a-0). This procedure was adapted from the literature.[1] 2-Bromopropionic acid (5.0 g, 32.7 mmol) was dissolved in CH_2Cl_2 (40 mL). *N*,*N*-Dicyclohexylcarbodiimide (3.745 g, 18.1 mmol) was added, and the milky mixture was stirred overnight at room temperature. After reaction, the precipitate was filtered off and the filtrate was concentrated using rotary evaporation. The concentrated solution was poured in *n*-pentane, thus resulting in the precipitation of insoluble species. The residue was filtered, and the filtered solution was concentrated *in vacuo*. The resulting slightly greenish liquid was obtained in 56% yield. ^{1}H NMR (400 MHz, $CDCl_3$): δ (ppm) = 1.83 (q, 6H, -CO-CH-$\underline{CH_3}$), 4.39 ppm (m, 2H, -CO-$\underline{CH}$-CH_3). ^{13}C NMR (100 MHz, $CDCl_3$) δ 20.84, 39.22, 164.42.

Model reactions. In order to optimize the coupling conditions between an acyclic acid anhydride and a primary amine, model reactions were performed and monitored *in situ* by ^{1}H NMR (Supplementary Figure 1a). Two different anhydrides, 2-bromopropionyl anhydride and 2-bromoisobutyryl anhydride, were reacted with cyclohexylamine. The latter cyclic compound was selected to mimic 4-amino-TEMPO. In all cases, 30 mg of anhydride (2 Eq.), requisite quantity of amine (1 Eq.), K_2CO_3 (3 Eq.) along with 0.5 mL of CD_3CN was taken in an NMR tube. Directly after addition of the components, a first ^{1}H NMR spectrum was recorded (Supplementary Figure 1b-c). In both model reactions, the methine proton of cyclohexylamine adjacent to the amine group at 2.5 ppm (proton **a**) fully vanished and were replaced by new peaks around 3.5 ppm corresponding to a methine proton adjacent to an amide bond (proton **a'** and **a"** in Supplementary Figures 1b-c). These results indicate that the amidification was quantitative during the time interval needed for the tube preparation and NMR analysis (i.e. about a few minutes). Moreover, the analysis of the raw experimental mixtures by LC-MS confirmed the formation of the amide adducts. ESI-LCMS was carried out on a Waters Acquity UPLC-SQD apparatus equipped with a PDA detector (190-500 nm, 80Hz), using a reverse phase column (Waters, BEH C18 1.7 mm, 2.1mm x 50 mm) and the MassLynx 4.1 – XP software. The mobile phase was methanol/water with 0.005% ammonia, and a solution of the organic compound in methanol was prepared for the analysis. ESI-MS: m/z calculated for $[M+H]^+$ $C_9H_{17}BrNO$ (2-bromo-*N*-cyclohexylpropanamide): 234.04, found 234.00; m/z calculated for $[M+H]^+$ $C_{10}H_{19}BrNO$ (2-bromo-*N*-cyclohexyl-2-methylpropanamide): 248.05, found 248.097. The formation of di-substituted imides could not be detected by mass spectrometry.

Oligomer synthesis on the glycine-loaded Wang soluble polystyrene S2. The following examples describe the successive coupling of **1** and amino-TEMPO to the glycine-loaded Wang soluble polystyrene support **S2** (M_n = 5700 g·mol^{-1}; M_w/M_n = 1.10) and can be understood as a general procedure for the synthesis of poly(alkoxyamine amide)s on a cleavable soluble support. Steps E2 and E3 can be repeated a certain number of times in order to reach an oligomer of desired length.

Removal of the Fmoc-group. 0.6 g of **S2** was dissolved in a mixture of piperidine/DCM (1/1, 6 mL) and the solution was stirred for 1.5h at room temperature. The reaction mixture was concentrated under reduced pressure and precipitated in methanol. The precipitate was collected by filtration, washed with methanol and dried in vacuum.

Attachment of the 1-motif to the soluble support S2. The amine terminated polymer **S2** (0.50 g, 1 Eq.) along with **a-1** (0.166 g, 6 Eq.) and K_2CO_3 (0.181 g, 15 Eq.) were dissolved in 5mL of THF. The solution was stirred for 50 min at room temperature. After reaction, the solvent was removed under reduced pressure. The resulting white solid was dissolved in THF and filtered. The filtrate was concentrated under reduced pressure and precipitated in MeOH. The precipitate was filtered, washed with MeOH and dried.

Attachment of amino-TEMPO to the soluble support. The bromine-functionalized soluble support (0.47 g, 1 Eq.), amino-TEMPO (0.090 g, 6 Eq.) and Me_6TREN (0.15 mL, 6.6 Eq.) were dissolved in a mixture of dry THF and DMSO (1:1 v/v) and placed in a round-bottom flask sealed with a rubber septum. The solution was then purged with argon for about 15 min. Then, CuBr (0.050 g, 4 Eq.) was rapidly added. The resulting solution was stirred for 30 min at room temperature. After completion, the reaction mixture was concentrated under reduced pressure, precipitated in MeOH and the precipitate was collected by filtration, washed with methanol and dried under vacuum.

Cleavage of the oligomers from soluble support. Cleavage of the poly(alkoxyamine amide)s from the soluble support **S2** was performed in TFA/DCM solution (1/1) for 2 h. After completion, the resulting solution was concentrated and precipitated in cold methanol. The oligomers along with small quantity of polymer support were isolated from the filtrate by removing the solvent and TFA. Pure oligomers were obtained by dissolving the residual solid in diethyl ether and filtration. Example of final yield: (For Heptamer) 20 mg from 0.5 g of **S2**; yield = 24%.

Oligomer synthesis on the non-cleavable polystyrene soluble-support S3. The following examples describe the successive coupling of **1** and amino-TEMPO to the soluble polystyrene support **S3** (M_n = 7460 g·mol^{-1}; M_w/M_n = 1.17) and can be understood as a general procedure for the synthesis of poly(alkoxyamine amide)s on a bromine-terminated soluble support. Steps F1 and F2 can be repeated a certain number of times in order to reach an oligomer of desired length.

Attachment of amino-TEMPO to the soluble support. Soluble support **S3** (1.8 g, 1 Eq.), amino-TEMPO (0.23 g, 3 Eq.), CuBr (0.084 g, 1.3 Eq.) and Me_6TREN (0.14 mL, 1.3 Eq.) were dissolved in a mixture of dry THF and DMSO (2:1 v/v) and placed in a round-bottom flask sealed with a rubber septum. The solution was then purged with argon for a few minutes and stirred for 15 min. The polymer was precipitated in cold MeOH and the precipitate was collected by filtration, washed with methanol and dried in a vacuum oven for 2h.

Attachment of the 1-motif to the soluble support. The amino-TEMPO-loaded soluble support (1.75 g, 1 Eq.) was mixed with **a-1** (0.69 g, 5 Eq.) in a solution of DIPEA (1.75 mL, 23 Eq.) and anhydrous DCM (4 mL). The mixture was stirred for 50 min and the polymer was then precipitated in MeOH. The precipitate was collected by filtration, washed with methanol and dried in a vacuum oven for 2h.

Sequencing. As illustrated in Figure 1c, studied species are terpolymers composed of the two coding units **0** ($m_{\mathbf{0}}$ = 71 Da) and **1** ($m_{\mathbf{1}}$ = 85 Da) separated by an alkoxyamine spacer T (m_T = 155 Da), and holding an acetyl moiety as the α-end-group (m_α = 59 Da) and Br as the ω-end-group (m_ω = 79/81 Da). Collisional activation of these species electrosprayed as protonated molecules induces a main dissociation mechanism, which consists of the homolytic cleavage of the C– O bond between a coding monomer and the spacer. Depending on the location of the adducted proton relative to the reacting center, such a charge-remote cleavage leads to the formation of a protonated nitroxide or a protonated carbon-centered radical (*vide infra*). Since the nature of coding monomers does not influence the mechanism of this homolytic cleavage, the nomenclature established for product ions of synthetic polymers can be usefully employed to simplify designation of these product ions in MS/MS spectra.[2] For this purpose, studied species have to be considered as copolymers composed of **0**-T and **1**-T units, the α-end-group and either **0**-ω or **1**-ω as a terminal motif. Considering the latter structural definition,

designation of product ions can be done according to the nomenclature established by Wesdemiotis *et al.* as briefly described hereafter.[2] Protonated nitroxides are named “y” since they contain the original ω-end-group (hence designated by a letter from the end of the alphabet) and they are formed after the cleavage of the second bond in the monomer (when counting bonds in the skeleton from right to left). In contrast, protonated carbon-centered radicals contain the original α-end-group (hence designated by a letter from the beginning of the alphabet) and are formed after the cleavage of the third bond in the monomer (when counting bonds in the skeleton from left to right): they are then named “c”. For both product ions, a superscripted “•+” is added to indicate that they are radical cations, and the subscripted “i” value corresponds to the number of partial or entire motifs (**0**-T or **1**-T) that they contain.

Using this nomenclature to annotate peaks in MS/MS spectra, interpretation of CID data can readily be performed based on relative locations of **1** and **0** in the precursor ion, according to the rules described below:

Rule 1. The largest congener in the $c_i^{\bullet+}$ product ion series (*i.e.*, $c_n^{\bullet+}$) is formed after the precursor ion has eliminated either a 305 Da radical ($^{\bullet}$T-**0**-ω) when containing the **0**-ω moiety, or a 319 Da radical ($^{\bullet}$T-**1**-ω) when containing the **1**-ω moiety.

Rule 2. The largest congener in the $y_i^{\bullet+}$ product ion series (*i.e.*, $y_n^{\bullet+}$) is formed after the precursor ion eliminates either a 130 Da radical (α-**0**$^{\bullet}$) when containing the α-**0** moiety, or a 144 Da radical (α-**1**$^{\bullet}$) when containing the α-**1** moiety.

Rule 3. The additional coding unit present in $c_i^{\bullet+}$ compared to $c_{i-1}^{\bullet+}$ (as well as in $y_i^{\bullet+}$ compared to $y_{i-1}^{\bullet+}$) is revealed by the *m/z* difference ($\Delta m/z$) between these two product ions and is a **0** if $\Delta m/z$ = 226 Da or a **1** if $\Delta m/z$ = 240 Da.

As a result, measuring the mass of the two smallest neutrals released from the precursor ions allows the highest congeners of each product ion series to be identified, and so the coding unit linked to each termination. Then, measuring the distance between consecutive peaks from $c_n^{\bullet+}$ down to $c_2^{\bullet+}$ and from $y_n^{\bullet+}$ down to $y_2^{\bullet+}$ allows the binary (**1**, **0**) sequence in the precursor ion to be reconstructed, starting from the ω– or α- chain-end, respectively. However, it is important to note that the smallest congeners in each series (i.e., $c_1^{\bullet+}$ and $y_1^{\bullet+}$) were never observed. As shown in Figure 3b-c, isomers composed of one **0** and two **1** coding units can readily be distinguished when applying these sequencing rules. Longer sequences can also easily be deciphered as shown in Figure 1e.

Supplementary references.

1 Östmark, E., Harrisson, S., Wooley, K. L. & Malmström, E. E. Comb Polymers Prepared by ATRP from Hydroxypropyl Cellulose. *Biomacromolecules* **8**, 1138-1148 (2007)

2 Wesdemiotis, C. *et al.* Fragmentation pathways of polymer ions. *Mass Spectrom. Rev.* **30**, 523-559 (2011)
